# Gender, Cognitive Status, and Depressive Symptoms: Findings From the National Social Life, Health, and Aging Project

**DOI:** 10.1093/geroni/igaa057.203

**Published:** 2020-12-16

**Authors:** Ethan Siu Leung Cheung, Ada Mui

**Affiliations:** 1 Columbia University, Bronx, New York, United States; 2 Columbia University, New York, New York, United States

## Abstract

This secondary research is based on the Wave 3 National Social Life, Health and Aging Project (n = 3,104). The association between cognition, gender, and depressive symptomatology were examined. Findings indicate that 54% of the sample were women and the mean age was 72.95 (SD=8.29). Bivariate analyses suggest that there were no gender differences in cognitive status (Mean of MoCA Short Form = 9.73; SD = 3.26), age, and stress (Mean of PSS = 7.69; SD = 3.90). There were significant gender differences in terms of marital status, income, education, stressors, social participation, and social support. Compared to older men, older women reported a significantly lower level of both education and income. Multiple regression results show that gender has an independent effect and a joint effect with stressors in explaining depressive symptoms. Parallel regression analyses for each gender group were conducted and models were significant (P < .0001). The only common predictor for depressive symptoms was ADL impairment, and the impact of this was stronger for males (b=.32) than for females (b=.17). For older men, unique correlates of depressive symptoms were being not married, more ADL and cognitive impairments, and higher stress. For older women, a higher level of depressive symptoms was associated with being younger, lower-income, a higher level of ADL and IADL impairments. In addition, white elderly women reported a higher level of depressive symptoms than Asian elderly women. Findings suggest gender and racial differences in depressive symptoms experienced among older Americans living in the community.

